# Supplementary material for: Identification of photoperiod-induced specific miRNAs in the adrenal glands of Sunite sheep (Ovis aries)
Source: Front Vet Sci. 2022 Jul 22;9:888207. doi: 10.3389/fvets.2022.888207 (PMC9354845; doi:10.3389/fvets.2022.888207)
Supplement: Supplementary file 1 [file Data_Sheet_1.ZIP › Supplementary file/miRDeep2 file.zip!/miRDeep2 file/Know_Structure/SPLP42A1/oar-mir-3958.pdf]

miRBase precursor : oar-mir-3958
Total read count : 12579
oar-miR-3958-5p read count : 11
oar-miR-3958-3p read count : 12565
remaining reads : 3

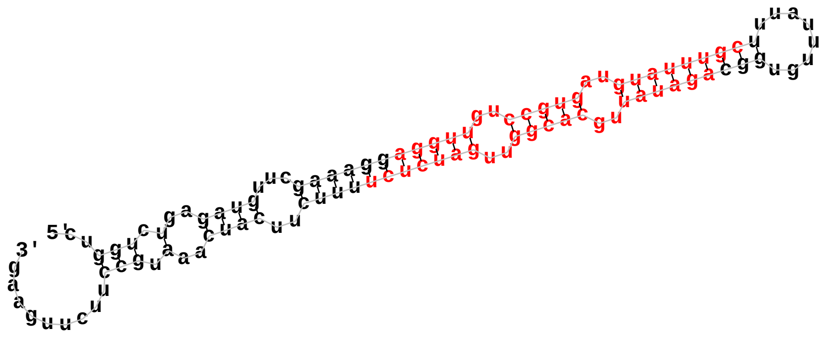

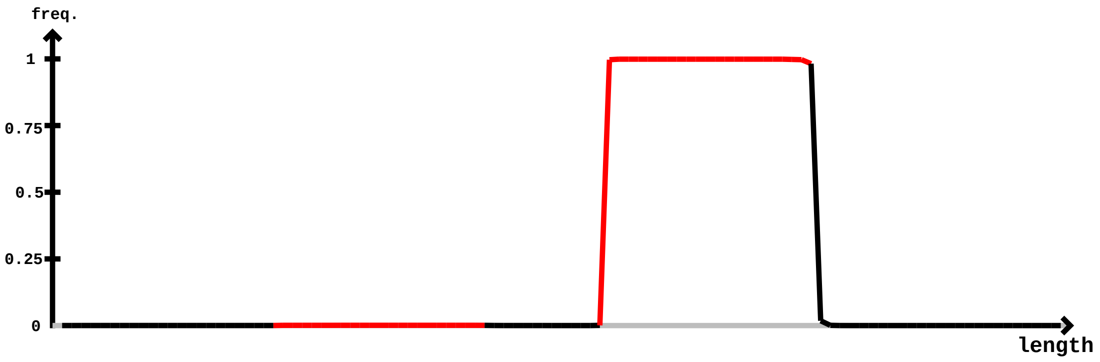

oar-miR-3958-3p

oar-miR-3958-5p

```
5'- cuggucugagauguucgaaaggagguuguccgugauguauuugcuuuauuuguggcagauauugcacgguugaucucuuuucuucaucaaaugccuucuugaag  -3'  exp
    ..(((.(..((((...((((((((((..(((((..((((((((........))))))))..)))))..))))))))))..))))..).))).............  reads  mm  sample
    cuggucugagauguucgaaagg..................................................................................  1      0   seq
    .uggucugagauguucgaaagg..................................................................................  1      0   seq
    ......................agguuguccgugauguauuug.............................................................  2      0   seq
    ......................agguuguccgugauguauuugc............................................................  6      0   seq
    ......................agguuguccgugauguauuugcu...........................................................  3      0   seq
    ............................uccgugauguauuugcuuuauu......................................................  1      0   seq
    .......................................................cagauauugcacgguugaucucu..........................  10     0   seq
    ........................................................agauauugcacgguugauc.............................  13     0   seq
    ........................................................agauauugcacgguugaucu............................  11     0   seq
    ........................................................agauauugcacgguugaucuc...........................  189    0   seq
    ........................................................agauauugcacgguugaucucu..........................  12089  0   seq
    ........................................................agauauugcacgguugaucucuu.........................  216    0   seq
    ........................................................agauauugcacgguugaucucuuu........................  10     0   seq
    ........................................................agauauugcacgguugaucucuuuu.......................  1      0   seq
    .........................................................gauauugcacgguugaucucu..........................  26     0   seq
```
